# Epigenetic Alterations of Heat Shock Proteins (HSPs) in Cancer

**DOI:** 10.3390/ijms20194758

**Published:** 2019-09-25

**Authors:** Hyun Seung Ban, Tae-Su Han, Keun Hur, Hyun-Soo Cho

**Affiliations:** 1 Korea Research Institute of Bioscience and Biotechnology, Daejeon 34141, Korea; 2 Department of Biochemistry and Cell Biology, School of Medicine, Kyungpook National University, Daegu 41944, Korea

**Keywords:** HSPs, epigenetics, miRNA, DNA methylation, histone methylation, cancer

## Abstract

Heat shock proteins (HSPs) are associated with various physiological processes (protein refolding and degradation) involved in the responses to cellular stress, such as cytotoxic agents, high temperature, and hypoxia. HSPs are overexpressed in cancer cells and play roles in their apoptosis, invasion, proliferation, angiogenesis, and metastasis. The regulation or translational modification of HSPs is recognized as a therapeutic target for the development of anticancer drugs. Among the regulatory processes associated with HSP expression, the epigenetic machinery (miRNAs, histone modification, and DNA methylation) has key functions in cancer. Moreover, various epigenetic modifiers of HSP expression have also been reported as therapeutic targets and diagnostic markers of cancer. Thus, in this review, we describe the epigenetic alterations of HSP expression in cancer cells and suggest that HSPs be clinically applied as diagnostic and therapeutic markers in cancer therapy via controlled epigenetic modifiers.

## 1. Introduction

Heat shock proteins (HSPs) are conserved molecular chaperones that are divided into small HSPs, including HSP40, HSP60, HSP70, HSP90, and HSP110, according to their molecular weight [1,2,3]. In cells, HSPs inhibit misfolded proteins in response to cellular stress, such as high temperature and hypoxia [4]. HSPs are also associated with the apoptotic machinery via caspase-dependent or caspase-independent pathways and the immune response because they are expressed on the cell surface [5]. Recently, several studies have reported that HSP expression is maintained at high levels in cancer (due to overproduction of oncoproteins, hypoxia, and genomic instability) and that HSPs are critically involved in cancer cell proliferation, metastasis, invasion, and angiogenesis [3,6,7]. Thus, HSPs are recognized as therapeutic and diagnostic targets for cancer treatment. To regulate HSP expression, various regulatory proteins participate in the transcription and translation of HSPs. Among these proteins, proteins involved in the epigenetic machinery (involving histone modifications as well as miRNA and DNA methylation) are important regulators of gene expression. Additionally, in cancer, epigenetic modifiers are overexpressed and associated with the regulation of oncogenes, tumor suppressor genes, and HSPs by epigenetic regulation (e.g., hypo/hypermethylation in DNA, mRNA degradation by miRNA, and euchromatin/heterochromatin in histones) [8,9,10,11,12,13,14,15].

Thus, in this review, we introduce HSPs and the epigenetic regulation of HSP expression in cancer and suggest their clinical application for cancer treatment. Additionally, we propose the development of anticancer drugs for the regulation of HSP expression based on our understanding of epigenetic alterations.

## 2. Epigenetic Alterations of HSPs Expression in Cancer

### 2.1. HSP60

HSP60 is a mitochondrial stress chaperone that is mainly located in eukaryotic mitochondria and involved in protein folding, cell apoptosis, and responses to cellular stress [3]. Moreover, in the extracellular space and at the cell surface, HSP60 serves as an antigen for T and B lymphocytes in the immune system [16]. In cancer, HSP60 expression associates with cell survival and apoptosis by regulating caspase activation [3] and with cancer metastasis by interacting with ß-catenin [17]. Regarding its utilization as a diagnostic marker for cancer, HSP60 expression levels are increased in several types of cancer and are associated with poor prognosis [3]. The epigenetic machinery (especially miRNAs and histone modification) can regulate HSP60 expression during cancer progression and metastasis. Additionally, the translational modification of HSP60 (e.g., through acetylation) has been observed in several cancers (Table 1).

#### 2.1.1. miRNAs and DNA Methylation of HSP60

High serum levels of miR-29a were found in breast cancer patients. The function of miR-29a was verified in MCF-7 breast cancer cells, revealing that its knockdown induced apoptosis in MCF-7 cells by upregulating HSP60 expression [18]. In addition, regarding HSP60 regulation by DNA methylation, the exposure of pigeons to avermectin (AVM) reduced DNA methyltransferase (DNMT) expression and induced HSP60 transcription in cardiac tissue [19]. However, HSP60 regulation by DNA methylation in cancer has not yet been reported.

#### 2.1.2. Histone and Translational Modifications of HSP60

In clinical breast cancer cohorts, EHMT2 (Euchromatic histone-lysine N-methyltransferase 2), a histone methyltransferase, was overexpressed, and EHMT2 knockdown upregulates the expression of HSP60, thus inducing cell apoptosis [11]. Regarding the translational modification of HSP60, after treatment with SAHA (Suberoylanilide hydroxamic acid), a histone deacetylase inhibitor (HDACi), HSP60 nitration and secretion were shown to be induced via SAHA-induced mitochondrial dysfunction and reactive oxygen species (ROS) overproduction, resulting in G2/M phase arrest in lung cancer cells [20]. Moreover, doxorubicin treatment induced HSP60 acetylation, and acetylated HSP60 inhibited p53 formation, resulting in p53-dependent senescence in lung cancer cells [21]. In osteosarcoma (OS), treatment with geldanamycin (GA), a novel inhibitor of OS, induced HSP60 expression and cancer cell apoptosis by reducing HSP60 hyperacetylation in mitochondria [22].

### 2.2. HSP70

HSP70 family members are molecular chaperones that play key roles in cellular processes by regulating protein folding [23]. In mammalian cells, various isoforms have been reported, including inducible HSP70 (HSPA1A and HSPA1B), constitutively expressed HSC70/HSP73/HSPA8, mitochondrial HSPA9/mortalin, and endoplasmic reticulum-resident HSPA5/glucose-regulated protein 78 (GRP78)/BiP. These chaperones are associated with human diseases such as cancer [24], inflammation [25], and Alzheimer’s disease [26], indicating that the HSP70 family may be a therapeutic target for multiple diseases. This section provides information on regulation of the HSP70 pathway by epigenetic modifications (Table 2).

#### 2.2.1. DNA Methylation of HSP70

Hypermethylation of the HSPA1A promoter has been reported in human ovarian and bladder cancer cells [27]. UM-UC10 and UM-UC13 bladder cancer cells express HSPA1A mRNA at a very low level, and treatment with the DNA methyltransferase inhibitor 5-aza-2′-deoxycytidine (Aza) restored HSPA1A expression [28]. Furthermore, methylation-specific PCR revealed that decreased HSPA1A expression is associated with the promoter methylation status.

In addition to the regulation of HSP70 expression by DNA methyltransferases through epigenetic modifications, a DNA methylation-independent mechanism of HSP70 regulation has also been reported. In human lung adenocarcinoma A549 cells, the knockdown of DNMT1 induced the expression of HSPA2 [29]. The mechanism by which DNMT1 reduces HSPA2 expression was shown to involve either direct or indirect interaction with the Sp1 protein rather than DNA methylation or histone acetylation [29]. Furthermore, indirect epigenetic regulation of the HSP70 chaperone axis has been revealed in hepatocellular carcinoma (HCC). Jiang et al. demonstrated that reduced HBP21 expression in HCC tissues by methylation of its promoter was correlated with short overall survival [30]. HBP21 acts as a cochaperone of HSP70 and inhibits the interaction between HSP70 and Bax, which results in the translocation of Bax from the cytoplasm to mitochondria and the induction of apoptosis [30].

#### 2.2.2. miRNAs of HSP70

In pancreatic cancer cells, miR-142-3p suppressed HSP70 expression by binding to its 3′ untranslated region (UTR), and overexpression of HSP70 reduced miR-142-3p-induced cell death, indicating that miR-142–3p is a negative regulator of HSP70 [31]. The well-known triptolide quercetin inhibited proliferation and HSP70 expression by upregulating miR-142–3p in pancreatic cancer cell lines [31]. HSP70 was overexpressed in OS and involved in chemoresistance. In OS, miR-233 negatively regulated the HSP70 protein levels by binding to the HSPA1A 3′-UTR, indicating that miR-233 might target HSP70 to regulate the chemoresistance of OS [32].

Several studies have reported the indirect regulation of HSP70 activity by miRNAs in cancer. In HCC, HSPA1B is a key regulator that promotes proliferation and blocks apoptosis by interacting with ATF7 (Cyclic AMP-dependent transcription factor 7). In Huh7 cells, miR-340-5p suppresses ATF7 expression by binding to its 3′-UTR, resulting in the induction of apoptosis and inhibition of proliferation through the miR-340-5p/ATF7/HSPA1B axis [33]. In esophageal squamous cell carcinoma (ESCC), miR-202 acts as a tumor suppressor by regulating HSF2 and its target gene HSP70 [34]. Overexpression of HSF2 suppressed miR-202-induced cell apoptosis by enhancing HSP70 expression in ESCC EC9706 and KYSE-510 cells [34].

#### 2.2.3. Histone Modifications of HSP70

Histone methylation is another epigenetic modification that regulates HSP70 expression. In human oral squamous cell carcinoma cell lines, heat treatment enhanced HSP70 expression by inducing the methylation of histone H3 Lys4 and H3 Lys9 [35]. The HSP inhibitor KNK437 suppressed heat-mediated HSP70 expression by inhibiting H3 Lys4, but not H3 Lys9 methylation [35]. Another study on the regulation of HSP70 expression by histone modification aimed to clarify the mechanism of action [36]. In Drosophila, heat shock induced recruitment of the TAC1 (Tachykinin, Precursor1) histone modification complex to the 5′-coding region of the HSP70 gene, which resulted in the modification of nucleosomes, specifically those that methylate and acetylate histone H3 [36].

#### 2.2.4. Translational Modification of HSP70

Lysine methylation has been suggested to play a role in the function of the HSP70 protein in various studies. Cho et al. reported that the methylation of HSP70 at Lys561 was elevated in various tumors relative to that in normal tissue and that methylated HSP70 directly interacted with and enhanced the kinase activity of Aurora kinase B [37]. Jakobsson et al. identified METTL21A as an enzyme responsible for the methylation of lysine residues in human HSP70 proteins [38]. In addition, methylation of HSPA8 reduced its affinity for soluble α-Syn, whose aggregation is associated with Parkinson’s disease [38]. Proteomic analysis of lysine methyltransferases by Shimazu et al. also clarified that METTL21A methylates HSP70 family proteins, including HSP70, GRP75, GRP78, and HSP7C [39].

### 2.3. HSP90

HSP90 is an essential molecular chaperone in eukaryotes and was first identified in complex with the oncoprotein viral Src kinase (v-Src) and steroid hormone receptors [40,41]. Evolutionarily, HSP90 is a conserved chaperone molecule that is involved in activating and stabilizing over 200 targets [42,43]. HSP90 targets are involved in important cellular processes associated with cell growth and proliferation. HSP90 possesses an ATP-binding cleft for protein kinase, and its dimer form is essential for its chaperone function.

In general, HSP90 is expressed at low levels in normal cells, but its expression is increased under stress conditions to protect cellular proteins from damage or aggregation of its targets. However, HSP90 is frequently upregulated in several cancer types, such as prostate cancer, leukemia, and lung cancer, and promotes tumor cell adhesion, motility, and metastasis by stabilizing and activating its targets, many of which are oncogenes, including downstream gene regulators, transcription factors, and kinases [44]. In this section, we will discuss the regulatory mechanisms of HSP90 caused by epigenetic modifications in cancer (Table 3).

#### 2.3.1. miRNAs of HSP90

Several studies have shown that miRNAs directly regulate HSP90 by targeting its 3′-UTR. Li et al. found that HSP90B1 (glucose-regulated protein 94, GRP94), a member of the HSP90 family, is regulated by miR-223 in OS [45]. The authors also showed that the overexpression of miR-223 suppressed HSP90B1 expression, whereas the inhibition of miR-223 increased HSP90B1 expression. The negative regulation of HSP90B1 by miR-223 significantly inhibited cell growth and promoted apoptosis through the PI3K/Akt/mTOR signaling pathway. Therefore, miR-223 plays a tumor suppressive role in OS. Another study investigated the relationship between miR-628-3p and HSP90 in A549 lung cancer cells [46]. Using bioinformatics algorithms and luciferase reporter assays, the authors identified HSP90 as a target gene of miR-628-3p, which functions as a tumor suppressor by promoting apoptosis and inhibiting the migration of A549 cells through negative regulation of HSP90. Choghaei et al. reported that suppressing miR-29a by an inhibitor broadly decreased the expression of HSP family members, including HSP27, HSP40, HSP70, and HSP90, via an indirect pathway in MCF-7 breast cancer cells [18]. The downregulation of HSP90 by anti-miR-29a induced sensitization to the anticancer drug Taxol.

p23, a cochaperone of HSP90, is an essential component of the HSP90 chaperone complex and inhibits ATPase activity by directly binding to the ATP-bound form of HSP90. p23 plays an important role in estrogen receptor (ER) alpha and telomerase activity, but its expression is upregulated in metastatic lung cancer, breast cancer, and prostate carcinoma. The overexpression of p23 in cancers promotes cell adhesion, invasion and lymph node metastases [47,48,49]. One group investigated the mechanism by which miRNAs regulate p23 in childhood acute lymphoblastic leukemia (ALL). In silico and in vitro experiments showed that p23 is negatively regulated by miR-101 [50]. Downregulated miR-101 was found in childhood ALL, and the dysregulation of miR-101 increased the p23 expression level.

#### 2.3.2. Translational Modification of HSP90

HSP90 activity is also altered by posttranslational modifications such as phosphorylation, methylation, and SUMOylation, which regulate HSP90 chaperone function [51]. A recent study showed that the mitotic checkpoint kinase Mps1 phosphorylates a threonine residue on HSP90, reducing its ATPase activity [52]. The phosphorylation of HSP90 is important for the mitotic checkpoint because it regulates the Mps1 stability and activity. Cdc14 is a phosphatase that dephosphorylates HSP90, and the phosphorylation of HSP90 increases its inhibitor sensitivity in renal cell carcinoma.

Previous studies have shown that SMYD2 is a histone H3K36 and H3K4 methyltransferase and functions as a transcriptional regulator [53,54]. However, SMDY2 is reportedly overexpressed in various cancer types and plays an important role in cell cycle regulation [55]. Recently, Hamamoto et al. found that SMYD2 methylates HSP90AB1 by interacting with the C-terminal region of HSP90AB1 and the SET domain of SMYD2 and regulates dimerization and chaperone complex formation [56]. The methylation of HSP90AB1 by SMYD2 promotes cancer cell proliferation.

HSP90α, an isoform of HSP90, has been reported to promote the extracellular maturation of MMP-2, which is involved in cancer cell invasion and metastasis [57,58]. Yang et al. found that the mechanism of HSP90α acetylation involves p300 as an acetyltransferase [59]. The authors also identified seven lysine residues on HSP90α using mass spectrometry, and HSP90α hyperacetylation was induced by treatment with the pan-HDAC inhibitor panobinostat (LBH589), an HDAC6 inhibitor, resulting in the attenuation of ATP binding and chaperone functions. Extracellular hyperacetylated HSP90α increases breast cancer cell invasion by binding to the MMP-2 chaperone. Therefore, these results suggest that anti-acetyl lysine-69 HSP90α can be used to diminish the invasiveness of breast cancer cells.

### 2.4. Others

#### 2.4.1. HSP27 (HSPB1, HSPB2)

The small HSP (HSP27), a small heat shock protein, assists in refolding misfolded proteins as a molecular chaperone and protects cells from heat shock and oxidative stress [60]. Increasing evidence has revealed the oncogenic roles of HSP27, which are associated with cell proliferation and drug resistance in many cancers [61]. Some studies have shown that miRNAs directly regulate HSP27 expression in several cancers. Yang et al. investigated whether miR-214 targeted HSP27, resulting in the inhibition of cell growth and sensitization to 5-FU in human colon cancer cells [62]. miR-577 also targets the HSP27 3′-UTR, leading to the suppression of HSP27. Overexpression of miR-577 increased 5-FU sensitivity and reduced xenograft tumor size in colorectal cancer [63]. Another study investigated whether miR-17–5p promotes migration through activating the p38 MAPK pathway and increasing the phosphorylation of HSP27. Thus, miR-17-5p functions as an oncogene in human HCC cells [64]. Another epigenetic modifier, DNA methylation, is associated with regulation of HSP27 expression. Promoter hypermethylation of the HSP27 gene was found in oral squamous carcinoma cells and ESCC, and its status was associated with the expression level of HSP27 [65,66]. Treatment with a DNA methyltransferase inhibitor (RG108) or 5-aza-2′-deoxy-cytidine led to an increase in the HSP27 expression level (Table 4).

#### 2.4.2. HSP40 Family (DNAJB6, MCJ)

DNAJB6 is a member of the HSP40 family, and it was reported to be a negative regulator of breast tumor progression. Mitra et al. identified miR-632 as an epigenetic regulator of DNAJB6 in breast cancer [67]. The miR-632 directly targets the coding region of DNAJB6. High expression of miR-632 leads to downregulation of DNAJB6 and increased cell invasion. In malignant pediatric brain tumors and epithelial ovarian cancer, the promoter methylation status of the MCJ gene was associated with disease pathogenesis and chemotherapeutic resistance [68,69] (Table 4).

#### 2.4.3. HSP110

Normally, HSP110 is induced by various stress conditions and promotes cell survival through its antiapoptotic and chaperone activities. A recent report demonstrated that HSP110 affects the Wnt/β-catenin pathway and has tumorigenic effects [70,71]. Another study investigated whether miR-27a expression suppresses the heat shock proteins HSP90 and HSP110 in human oral squamous cell carcinoma HSC-4 cells, resulting in decreased thermal resistance [72] (Table 4).

## 3. Clinical Relevance of HSPs in Cancer

HSPs are involved in various cancer development processes, such as cell proliferation, metastasis, invasion, and death, and are overexpressed in several types of cancer. Because HSP expression is associated with a poor prognosis and therapeutic resistance, the presence of HSP proteins may thus serve as diagnostic and prognostic cancer biomarkers as well as therapeutic targets. In addition, HSPs are released from cancer cells in their extracellular forms and can modulate malignant properties through receptor-mediated cellular signaling.

### HSPs as Cancer Treatment and Therapeutic Targets

**Figure 1:**
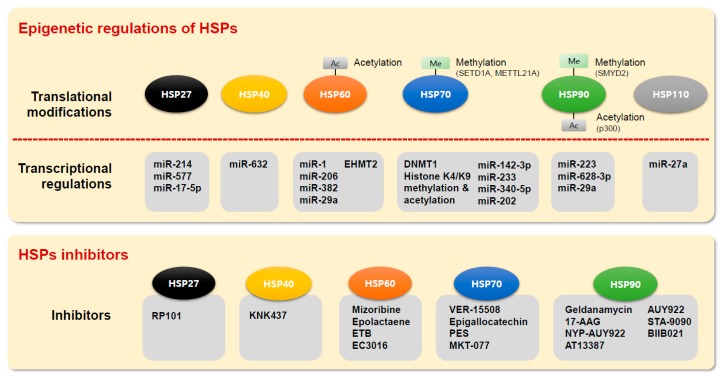
Summary for epigenetic regulations and inhibitors of HSPs in cancer. HSP27 inhibitor RP101 [87]; HSP40 inhibitor KNK437 [88]; HSP60 inhibitors mizoribine [89], epolactaene [90], ETB [90], and EC3016 [91]; HSP70 inhibitors VER-15508 [92], epigallocatechin [93], PES [94], and MKT-077 [95]; HSP90 inhibitors geldanamycin [96], 17-AAG [97], NYP-AUY922 [98], AT13387 [99], AUY922 [100], STA-9090 [101], and BIIB021 [102].

HSPs are important proteins in normal cells, but members of the HSP and small HSP families have been shown to be overexpressed in many cancers [73]. For example, overexpression of HSP60 has been observed in multiple cancers, such as glioblastoma [3,74], and HSP60 plays critical roles in metastasis, transformation, and angiogenesis through its antiapoptotic effects [3]. HSP70 overexpression has been associated with a poor prognosis in melanoma, cholangiocarcinoma, colon cancer, bladder cancer, breast cancer and uterine cervical cancer [3,24], and HSP70 is a survival factor that enhances carcinogenesis, is antiapoptotic in nature and is highly expressed in tumors [75]. Suppression of HSP90 expression led to the simultaneous coinhibition of several types of target proteins, thereby affecting multiple signaling pathways that suppress cell growth, apoptosis evasion, angiogenesis, invasion and metastasis [76,77,78,79]. Additionally, overexpression of HSP27 has been observed in acute myeloid leukemia, breast cancer, and oral squamous cell carcinoma [80,81,82], leading to the promotion of several aspects of carcinogenesis, including inhibition of apoptotic cell death and elevation of cytoprotection via direct interactions with several apoptotic proteins [83]. Although HSPs play critical roles in normal cells, HSP60, HSP70, and HSP90 are overexpressed in many cancers due to abnormal epigenetic regulation. In addition, cancer cells showed increased expression levels of HSPs as noted previously and also higher activity of HSPs relative to normal cells. HSP90 in cancer cells has a high ATPase activity that regulates chaperone function, whereas a latent form of HSP90 is present in normal cells [84,85]. Therefore, HSP90 inhibitors could effectively suppress the function of HSP90 with 20 to 200 times higher binding affinity in cancer cells than in normal cells [86]. Also, this evidence indicates that HSPs can be useful therapeutics for targeting cancers (Figure 1).

HSP27 is frequently increased by chemotherapy and has potent antioxidant and antiapoptotic properties [103,104]. HSP27 was overexpressed in gemcitabine-resistant human pancreatic cancer [105] and 5-fluorouracil (5-FU)-resistant colon cancer [106] and inhibited cisplatin-induced cell death through the unfolded protein response and autophagic activation in HCC [107]. For modulation of HSP27, small-molecule inhibitors, antisense oligonucleotides, and protein aptamers have been widely used [86,108,109]. Indeed, inhibition of the HSP27 and HSP40 proteins promoted the efficiency of 5-FU and carboplatin in hepatoma cells [110]. In addition, HSP27 suppression enhanced doxorubicin sensitivity and induced apoptosis in human colon carcinoma cells [111].

Similarly, HSP40 proteins also regulate the effects of chemotherapeutic agents. BMS-690514, a panHER/vascular endothelial growth factor receptor (VEGFR) inhibitor, promoted apoptosis by reducing the expression of HSP40 in erlotinib-resistant non-small cell lung cancer cells [112]. KNK437, a benzylidene lactam compound, inhibited the expression of HSP40 in human colon cancer cells [87].

HSP60 mediates drug resistance and could be a good drug target in cancer therapy. The inhibition of HSP60 has been reported to reverse drug resistance in 5-FU-resistant colorectal cancer cells [113]. In addition, GA-mediated cell death was correlated with the loss of mitochondrial HSP60 in OS cells, suggesting that HSP60 could be a good target of GA [21]. In melanoma cells, the inhibition of HSP60 expression by sinularin promotes the antitumor activities of cancer cells and is related to proapoptotic factors [114].

HSP70 proteins are also associated with mediating drug resistance in cancer therapy. Most malignant cells aberrantly express HSP70, which results in increased cell proliferation and malignancy. The HSP70 molecular chaperones, especially HSP72 and GRP78, are promising drug targets for cancer therapy. Fisetin, a dietary flavonoid, can induce the apoptosis of colon cancer cells by inhibiting the binding of HSF1 to the HSP70 promoter [115]. Similarly, cantharidin, a natural compound, induces cancer cell death via downregulating HSP70 and BAG3 by blocking the binding of HSF1 to promoters [116]. In liver cancer cells, apoptin inhibits HSP70 transcription and induces apoptosis by promoting HSF1 trimer depolymerization [117].

HSP90 plays a crucial role in cancer development and is thus a potential therapeutic target for cancer therapy. Currently, various HSP90 inhibitors have been investigated as cancer treatment agents in clinical trials. GA, a benzoquinone ansamycin antibiotic, binds to HSP90 and inhibits the cell migration associated with the downregulation of HIF-1α and phosphorylation of FAK in glioma cells via caspase-dependent apoptosis [118]. Tanespimycin, a GA derivative, induces apoptosis and cell cycle arrest in cancer cells by activating caspase-9 [119]. AUY922 (luminespib from Novartis), an HSP90 antagonist, suppresses in vitro cancer cell growth in a dose-dependent manner and is associated with a significantly reduced tumor volume (by 92% compared to that in untreated controls) [120].

Moreover, several HSP inhibitors are currently under clinical development, including STA-9090 (ganetespib from Synta), AT13387 (onalespib from Aztex), and IPI-504 (retaspimycin from Infinity), alone or in combination with other anticancer drugs. 

## 4. Conclusions

In this review, we describe HSP regulation via epigenetic alterations in cancer. MicroRNAs, DNA methylation, and histone modifications are involved in regulating the transcriptional HSPs level, and abnormal expression of HSPs by these epigenetic factors is associated with cancer development and malignant formation. Furthermore, activity of HSPs is regulated by post-translational modifications such as acetylation and methylation (Figure 1). Therefore, overexpressed HPSs and small HSPs are critically involved in regulating cell signaling pathways (e.g., PI3K/Akt/mTOR), ROS production, the cell cycle, and apoptosis in cancer cell proliferation. Moreover, the regulation of MMPs and several receptors (e.g., estrogen receptor) is controlled by overexpressed HPSs and small HSPs in cancer cell metastasis and invasion. Thus, understanding the epigenetic regulation of HSP expression can (1) facilitate the development of anticancer drugs using HSPs as therapeutic targets and (2) demonstrate synergistic anticancer effects between epigenetic modifiers and HSPs. Finally, accumulating evidence indicates that HSPs are crucial for advancing the accuracy of cancer diagnosis and developing effective chemotherapeutic targets.

## Figures and Tables

**Table 1:** Epigenetic regulations of heat shock protein 60 (HSP60) expression.

Name	Function
miR-1, miR-206	High-glucose-mediated apoptosis via HSP60 regulation
miR-382	Attenuation of renal interstitial fibrosis by the regulation of HSP60
miR-29a	Cell apoptosis by the regulation of HSP60 in MCF-7 breast cancer cells
EHMT2	Cell apoptosis by the regulation of HSP60 in MCF-7 breast cancer cells
HSP60 acetylation	Inhibition of the interaction between p53 and HSP60 in lung cancer
HSP60 acetylation	Induction of cell apoptosis via the hyperacetylated HSP60 mitochondrial protein in osteosarcoma

**Table 2:** Epigenetic regulations of HSP70 family member expression and activity.

Name	Function
HSPA1A promoter methylation	Decreased expression of HSPA1A by promoter methylation in bladder cancer cells
DNA methyltransferase 1 (DNMT1)/HSPA2	DNA methylation-independent regulation of HSPA2 expression through interaction with the Sp1 protein in lung cancer cells
HSP21 promoter methylation	Reduced expression of the HSP70 cochaperone HSP21 by promoter methylation in hepatocellular carcinoma cells
Histone H3 methylation	Heat-mediated HSP70 expression through histone H3 methylation in oral squamous carcinoma cells
Histone H3 methylation	Recruitment of TAC1 to the HSP70 promoter and methylation and acetylation of histone H3
HSP70 methylation	Enhancement of Aurora kinase B activity and cancer cell proliferation by methylated HSP70
HSP70 methylation	Reduction in the affinity for soluble α-Syn by methylated HSPA8
HSP70 methylation	Methylation of HSP70, glucose-regulated protein 75 (GRP75), GRP78 and HSP7C by the METTL21A methyltransferase
miR-142-3p	Regulation of pancreatic cancer cell proliferation by the suppression of HSP70 expression
miR-233	Negative regulation of HSP70 expression and modulation of chemoresistance in osteosarcoma
miR-340-5p	Regulation of proliferation by the miR-340–5p/ATF7/HSPA1B axis in HCC
miR-202	Regulation of HSF2-mediated HSP70 expression in esophageal squamous cell carcinoma

**Table 3:** Epigenetic regulations of HSP90 expression.

Name	Function
miR-223	Inhibition of cell growth and promotion of apoptosis by suppressing HSP90B1 in osteosarcoma
miR-628-3p	Tumor suppression by negative regulation of HSP90 in A549 lung cancer cells
miR-29a	Anti-miR-29a induces sensitivity to the anticancer drug Taxol by inhibiting HSPs, including HSP90
HSP90 methylation	SMYD2-mediated HSP90AB1 methylation promotes cancer cell proliferation
HSP90α acetylation	Extracellular hyperacetylated HSP90α increases breast cancer cell invasion

**Table 4:** Epigenetic regulations for HSP27, HSP40, and HSP110 expression.

HSPs	Name	Function
HSP27	miR-214	Inhibition of cell clone formation and cell growth and enhancement of cell apoptosis in colon cancer cells
	miR-577	Inhibition of cell growth and xenograft tumor growth and enhancement of 5-FU sensitivity in colorectal cancer cells
	miR-17-5p	Enhancement of cell migration in hepatocellular carcinoma cells through the p38 MAPK-HSP27 pathway
	HSP27 promoter methylation	Reduced expression level of HSP27 gene by promoter hypermethylation in oral squamous carcinoma cells and esophageal squamous cell carcinoma
HSP40	miR-632	Exogenous miR-632 expression increases the invasive ability by targeting DNAJB6 in breast cancer
	HSP40 promoter methylation	Epigenetic inactivation of MCJ gene by promoter hypermethylation is associated with disease pathogenesis in malignant pediatric brain tumor and epithelial ovarian cancer
HSP110	miR-27a	Increased hyperthermia-induced cell death in oral squamous cell carcinoma
